# Multiscale anisotropy analysis of second-harmonic generation collagen imaging of mouse skin

**DOI:** 10.1117/1.JBO.26.6.065002

**Published:** 2021-06-22

**Authors:** Karissa Tilbury, XiangHua Han, Peter C. Brooks, Andre Khalil

**Affiliations:** a University of Maine, Chemical and Biomedical Engineering, Orono, Maine, United States; b Maine Medical Center Research Institute, Scarborough, Maine, United States; c University of Maine, CompuMAINE Lab., Orono, Maine, United States

**Keywords:** anisotropy, second-harmonic generation, melanoma, cryptic epitope, wavelets

## Abstract

**Significance:** Morphological collagen signatures are important for tissue function, particularly in the tumor microenvironment. A single algorithmic framework with quantitative, multiscale morphological collagen feature extraction may further the use of collagen signatures in understanding fundamental tumor progression.

**Aim:** A modification of the 2D wavelet transform modulus maxima (WTMM) anisotropy method was applied to both digitally simulated collagen fibers and second-harmonic-generation imaged collagen fibers of mouse skin to calculate a multiscale anisotropy factor to detect collagen fiber organization.

**Approach:** The modified 2D WTMM anisotropy method was initially validated on synthetic calibration images to establish the robustness and sensitivity of the multiscale fiber organization tool. Upon validation, the algorithm was applied to collagen fiber organization in normal wild-type skin, melanoma stimulated skin, and integrin α10KO skin.

**Results:** Normal wild-type skin collagen fibers have an increased anisotropy factor at all sizes scales. Interestingly, the multiscale anisotropy differences highlight important dissimilarities between collagen fiber organization in normal wild-type skin, melanoma stimulated, and integrin α10KO skin. At small scales (∼2 to 3  μm), the integrin α10KO skin was vastly different than normal skin ($p\text{-value}∼10^{-8}$), whereas the melanoma stimulated skin was vastly different than normal at large scales (∼30 to 40  μm, $p\text{-value}∼10^{-15}$).

**Conclusions:** This objective computational collagen fiber organization algorithm is sensitive to collagen fiber organization across multiple scales for effective exploration of collagen morphological alterations associated with melanoma and the lack of α10 integrin binding.

## 1 Introduction

Collagen is the most abundant protein in our body, with estimates of at least 28 different subtypes identified as a primary protein component of the extracellular matrix (ECM). All collagen molecules are triple helical molecules characterized by a common tripeptide motif (Gly-X-Y) where the aminoacid glycine (Gly) resides in every third position. Collagen molecules are stabilized via hydrogen binding and are further stabilized into both fibril and fiber structures via covalent bounds.^1^^–^^3^ This unique structure of collagen provides a noncentrosymmetric organization of permanent dipole moments on the size scale of ∼300 to 500 nm,^4^ creating an ideal “harmonophore” for second-harmonic generation (SHG). SHG is governed by a nonlinear susceptibility tensor, which requires a permanent dipole moment with a noncentrosymmetric organization on the size-sale of the wavelength $\lambda_{\mathrm{SHG}}$.^5^ SHG imaging microscopy is a coherent process in which two photons interact with the noncentrosymmetric structure and create a single photon with exactly twice the frequency (half the wavelength, $\lambda_{\mathrm{SHG}}$) of the incident/excitation laser. Therefore, the physical underpinnings of SHG creation provide a unique label-free, collagen-specific imaging modality with sensitivity of the hierarchical organization of collagen spanning from molecular to supramolecular organizations.^5^^–^^7^ Given the sensitivity of SHG to collagen alterations and the prevalence of collagen in the ECM, SHG microscopy is frequently employed to quantify the dynamic remodeling of the ECM associated with both normal and abnormal processes. For example, SHG microscopy has been used to explore the natural remodeling of the collagen in the cervix during child-birth^8^^,^^9^ and the reorganization of collagen in the skin due to aging,^10^ as well as various diseased states including fibrotic diseases such as idiopathic pulmonary fibrosis^11^^,^^12^ and several distinct cancer types including breast, ovarian, pancreatic, colon, and melanoma.^13^^–^^19^

The role of collagen in the ECM is complex and multifaceted. Collagen is both a mechanical scaffold and an acellular regulator of cellular processes. The mechanical properties of collagen are tissue specific; however, desmoplasia, or increased collagen content and altered collagen alignment in tumor microenvironments, has been shown to be tumorigenic. There have been several publications highlighting the importance of SHG microscopy mapping both collagen features and collagen stiffness to tumor initiation, promotion, and metastasis in several epithelial cancers.^10^^,^^20^^–^^22^ In addition to these basic functions, collagen provides binding sites for a wide array of bioactive regulatory molecules including growth factors, cytokines, and other ECM proteins.^23^^–^^29^ Cellular interactions with collagen are dominated by integrins, a large family of multifunctional heterodimeric cell surface molecules.^24^^,^^25^^,^^29^ In particular, integrins α1β1, α2β1, α10β1, and α11β1 represent an important subgroup of collagen-binding integrin receptors^24^^,^^25^^,^^29^ that activate and coordinate different signaling cascades for unique tissue microenvironments.^24^^,^^25^^,^^29^ Recently, it was shown that integrin α10 is able to bind a unique cryptic region of collagen termed the HU177 epitope.^30^ This cryptic epitope is exposed during collagen degradation or remodeling associated with malignant breast, pancreatic, and ovarian carcinomas as well as malignant melanomas.^30^^–^^34^ Interestingly, cellular interactions with the HU177 cryptic collagen epitope can regulate the behavior of multiple cell types including melanoma cells.^30^^–^^34^ In animal models of melanoma, specific targeting of this cryptic collagen epitope reduced tumor growth and metastasis.^31^ While progress has been made in understanding how cellular interactions of α10β1 integrins with structurally intact collagen regulate cellular behavior, there is little known regarding whether integrin α10β1 plays a role in altering collagen structure or fiber orientation. To this end, experimental evidence from a mutant mouse model suggests that, while α10β1 integrin null mice are fertile with a normal life span, phenotypic alterations in bone development were observed.^35^ In addition to some bone abnormalities, the density of fibrillar collagen networks were also reduced, suggesting that the altered expression of α10β1 integrin may impact the deposition of collagen.^35^ Given these observations, developing new techniques, tools, and strategies to more precisely study and quantify collagen organization and architecture in biologically relevant tissue microenvironments is critical to translating the cellular and molecular understanding of the biophysical properties of collagen for developing highly innovative new applications for tissue engineering, imaging, and therapeutics. To this end, we provide a robust imaging and analytical framework perfectly suited to study the potential effects of integrins on collagen structure and formation.

SHG microscopy and computational image analysis are widely used to characterize tissue and disease-specific tissue remodeling. Unbiased, robust quantitative image analysis routines are essential tools. Currently, the quantitative analysis of collagen fiber features is broadly categorized into two types: (1) machine-learning/AI-based features for tissue classification and (2) physical collagen features. Machine-learning/AI-based feature extraction can be extremely powerful and has clearly identified relevant features for accurate classification in both 2D and 3D;^16^^,^^17^^,^^36^^–^^39^ however, these techniques frequently lack the connection to physical parameters. Therefore, these techniques are not viable when biophysical information of the tissue remodeling is desired. Alternatively, other computational analyses are focused on morphological features of collagen fibers in both 2D and 3D space. Fourier transform (FT)-based techniques characterize collagen fiber features in both 2D and 3D. Collagen fiber properties are derived from functional properties of the FT. The functional properties of the FT of an image can be evaluated through multiple approaches; several studies have used FT-based techniques to distinguish collagen fiber morphologies associated with breast and ovarian cancers.^40^^–^^44^ Recently, FiberFit, a fast, open-source FT-based method has gained attention in the biomechanics field as a robust quantitative tool of collagen alignment in ligaments.^45^ FT-based methods are useful; however, they frequently are not sensitive to the slight alterations frequently present in diseased tissues and are restricted to global analysis. Another simple quantitative analysis with FIJI plugins is the gray scale co-occurrence matrix (GLCM),^43^^,^^46^ which uses simple geometric relationships based on the intensity similarities of neighboring pixels. The GLCM and 2D FT methods have been combined into a support vector machine (SVM) classifier to describe ECM remodeling in a mouse model of ovarian cancer with ∼80% sensitivity and specificity.^43^ More complex methods for fiber alignment are based on curvelets, ridgelets, and wavelets. An adaptation of curvelets is provided in both curve align and CT-FIRE, which provide bulk and fiber-specific features such as fiber angle, fiber length, fiber width, etc. with respect to a tumor boundary.^36^ These techniques have been used in numerous publications involving breast, pancreatic, and ovarian cancer.^17^^,^^19^^,^^36^ The implementation of curvelets and the FT transform in open-source tools, such as curve align, CT-FIRE, and FiberFit, restricts the analysis to a single size-scale that potentially stymies the sensitivity of the tools to slight collagen alterations such as the morphological impacts of exposure of the integrin α10β1-binding cryptic collagen epitope. To address these limitations, we present an adaptation of the 2D wavelet transform modulus maxima (WTMM) anisotropy method. The 2D WTMM anisotropy approach uses a continuous wavelet for multiscale extraction of features, which provides greater sensitivity to slight morphological features of the collagen fibers. The 2D WTMM method was first introduced in the late 90’s as a generalization to the 1D WTMM method^47^^–^^50^ and is used for the multifractal analysis of selfsimilar (rough) surfaces. Its usage as a multifractal formalism^51^^–^^60^ and a segmentation technique^54^^,^^58^^,^^59^^,^^61^^–^^66^ in the applied sciences is widespread. The 2D WTMM anisotropy method was first spun off in 2006 for the investigation of the anisotropic signature of atomic hydrogen in the galactic plane.^56^ Since then, adaptations of the 2D WTMM anisotropy method have been used to study muscle cell morphology in zebrafish^67^^–^^71^ and soft tissue in-growth into artificial bone implants.^57^

In this paper, an adaptation of the 2D WTMM anisotropy is described and validated through a rigorous calibration on thousands of synthetic images of simulated fibers from a wide range of random alignments. After validation, the method is applied to characterize the multiscale morphological features of collagen fibers acquired using SHG microscopy from normal wild-type skin, skin stimulated with a melanoma tumor, and skin from integrin α10 knock-out animals (α10KO). We find that the multiscale 2D WTMM anisotropy method provides unique information regarding the morphological features of the collagen alterations and is therefore well-suited for the next frontier of quantifying the unique integrin/collagen impacts of tissue remodeling.

## 2 Materials and Methods

### 2.1 Cells and Cell Culture

B16F10 melanoma cells were obtained from ATCC (Manassas, Virginia) and cultured in DMEM medium with 10% FBS, 1% Pen-strep, and 1% sodium pyruvate.

### 2.2 Generation of Integrin α10 Knockout Mice

To generate integrin α10 knockout mice (α10KO), an integrin α10 targeting vector containing an ES cell clone in C57BL/6J background was developed by European Mouse Mutant Cell Repository.^72^ The integrin α10 gene function was inactivated by splicing of upstream endogenous exons to a splice acceptor in the targeting cassette located between exon7 and exon8. This allele reports gene expression by the lacZ-reporter and can function as a null mutation. The ES cell clone was expanded and microinjected into C57BL/6J blastocysts to generate a chimeric integrin α10 knockout founder line at the Mouse Transgenic & Gene Targeting Core of Maine Medical Center Research Institute (MMCRI). Genotyping PCR was performed using genomic DNA extracted from tail tips as templates. PCR primers used were common forward (5′-ACACACCTGTTCACTTCCCC-3′), wild-type reverse (5′-AAGGACCGCAATCCCATAAC-3′), and mutant reverse (5′-TAGAGTTCCCAGGAGGAGCC-3′). Homozygous integrin α10 knockout mice were born at the expected mendelian ratio and were viable and fertile without obvious gross abnormalities. Mice were housed in an MMCRI pathogen-free air barrier facility, and animal handling and procedures were approved by the MMCRI Animal Care and Use Committee.

### 2.3 Generation of B16F10 Melanoma Tumors and Control Tissues

Melanoma tumors were established essentially as previously described.^31^ Briefly, wild-type C57BL/6J mice (6 to 8 weeks old) were injected subcutaneously with $3.5\times 10^{5}$ B16F10 cells. Tumors were allowed to form for 14 days. At the end of the 14-day growth period, subcutaneous tumors with the associated skin were dissected and fixed with formalin. For nontumor stimulated mouse skin tissue preparation, wild-type C57BL/6J mice and integrin α10 knockout mice (α10KO) were shaved to remove hair and full thickness murine skin was dissected. All tissues were washed in PBS and fixed with formalin. Tissue sections were prepared from tumors or normal skin and stained by H&E.

### 2.4 Imaging

**Fig. 1:**
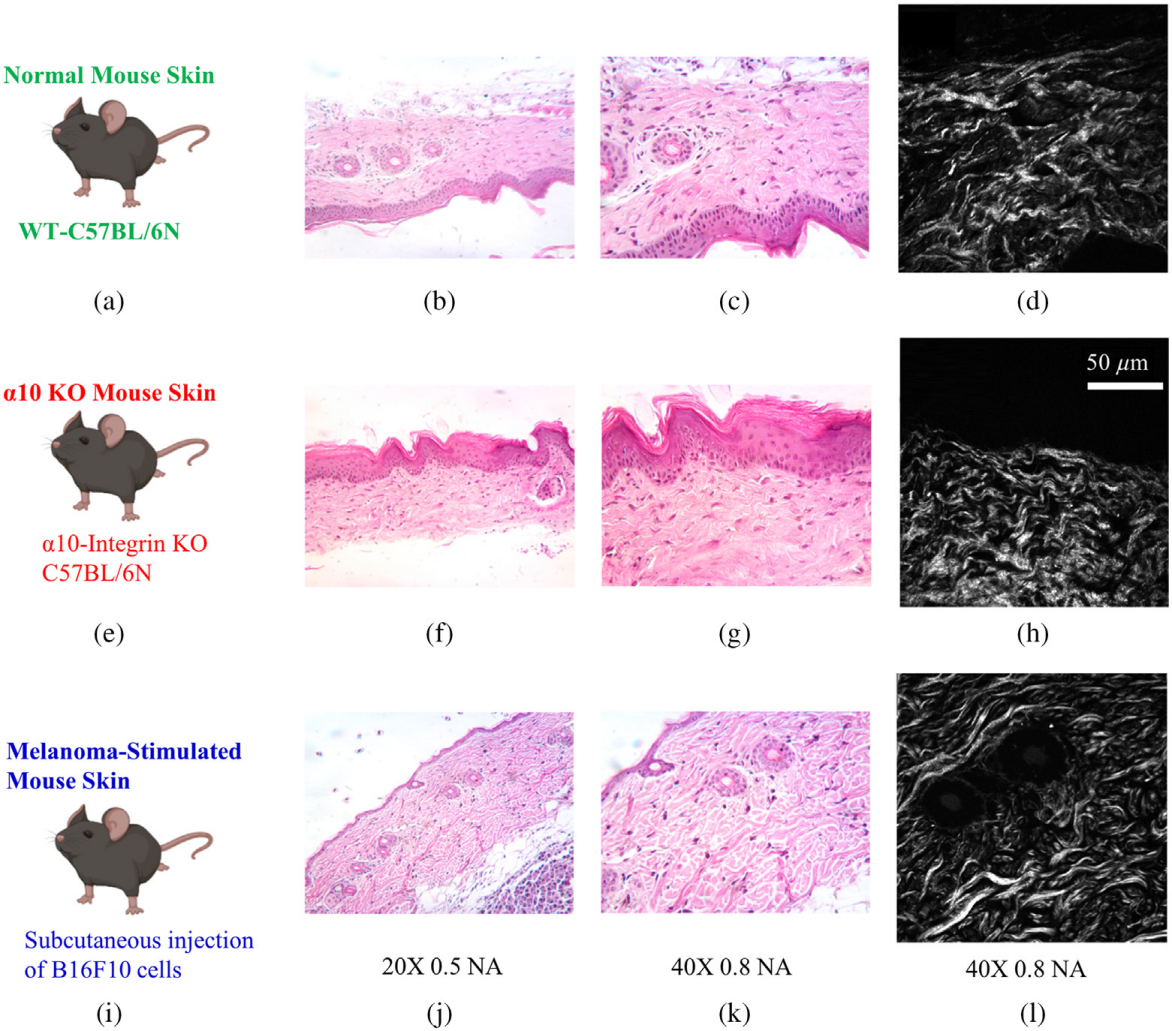
Sample bright-field and SHG images of the tissue samples. (a), (e), and (i) Cartoon depiction of the three mouse phenotypes. (b), (f), and (j) Tissue stained for H&E using the 20× objective; (c), (g), and (k) tissue stained using the 40× objective. SHG images 40× objective 2× optical zoom (selection shown in the middle column) of skin from (d) a normal wild-type mouse, (h) an alpha10 knockout, and (l) a mouse with melanoma.

SHG imaging of H&E-stained slides (10  μm) was performed using a custom-built two-photon microscope using an upright microscope stand (Olympus BX50WI, Olympus, Center Valley, Pennsylvania) with a laser scanning unit (Fluoview300, Olympus) that is coupled to a mode-locked titanium sapphire femtosecond laser (Chameleon Ultra II, Coherent, Santa Barbara, California). Laser power was modulated by an electro-optic modulator (ConOptics, Danbury, Connecticut), operated in a power range of 3 to 10 mW at the focal plane using a LUMPlanFLN 40× 0.8 NA (Olympus, Center Valley, Pennsylvania) water immersion objective. Circular polarization was used for SHG imaging; it was verified at the focal plane by rotating a polarizer and experiencing no change in laser power. All SHG imaging used 890-nm excitation, and the epi-SHG signal was collected using a 448/20-nm bandpass filter (Semrock Rochester, New York) in a nondescanned geometry using a H7421 GaAsP PMT (Hamamastsu, Hamamstsu City, Japan) using a LUMPlanFLN 40× 0.8 NA objective (Olympus, Center Valley, Pennsylvania) with 2× optical zoom (180  μm field of view) with a 512×512  pixels laser scanning speed of 2.71  s/frame with Kalman 4 averaging. Representative bright field images were acquired using a 5.1-MP MU 500 AmScope eyepiece camera using both LUMPlanFLN 20× 0.5NA and LUMPlanFLN 40× 0.8NA (Olympus, Center Valley, Pennsylvania) water immersion objectives. Figure 1 shows some sample bright-field images of H&E-stained slides from which SHG images were taken. Table 1 shows the number of mice used for each cohort and the number of individual XY SHG images taken from different regions of interest from each animal.

**Table 1:** Description of the number of animals and SHG images for each data category.

	Normal wild-type skin (control)	Melanoma-stimulated skin	α10 knockout normal skin
Number of mice	5	6	6
Number of images/mice	10	10	10
Total number of images	50	60	60

### 2.5 2D WTMM Anisotropy Method

In this implementation of the 2D WTMM method, the continuous wavelet-transform (WT), acting much like a “mathematical microscope,” is used to quantify image intensity fluctuations. Use of the continuous WT allows us to investigate a continuous range of size scales. The WT of an image is the gradient vector (strength and direction of largest intensity variation) of the image smoothed by dilated versions of a Gaussian (smoothing) filter. Specifically, consider the wavelets,^73^

$$\psi_{1}(x,y)=\frac{\partial \phi (x,y)}{\partial x}\;\text{and}\;\psi_{2}(x,y)=\frac{\partial \phi (x,y)}{\partial y},$$

where ϕ(x,y) is the 2D Gaussian (isotropic) smoothing function. The WT with respect to $\psi_{1}$ and $\psi_{2}$ is

$$T_{\psi }[f](b,a)=(\begin{array}{c} T_{\psi_{1}}[f]=a^{-2}\int d^{2}x\psi_{1}(a^{-1}(x-b))f(x)\\ T_{\psi_{2}}[f]=a^{-2}\int d^{2}x\psi_{2}(a^{-1}(x-b))f(x) \end{array})\;=T_{\psi }[f](b,a)=\nabla \{T_{\phi }[f](b,a)\},$$

from which we can get the modulus and argument (angle) of the WT:

$$T_{\psi }[f](b,a)=(M_{\psi }[f](b,a),\;A_{\psi }[f](b,a)),$$

where

$$M_{\psi }[f](b,a)={\left[{\left(T_{\psi_{1}}[f](b,a)\right)}^{2}+{\left(T_{\psi_{2}}[f](b,a)\right)}^{2}\right]}^{1/2},\;A_{\psi }[f](b,a)=\mathrm{Arg}(T_{\psi_{1}}[f](b,a)+iT_{\psi_{2}}[f](b,a)).$$

**Fig. 2:**
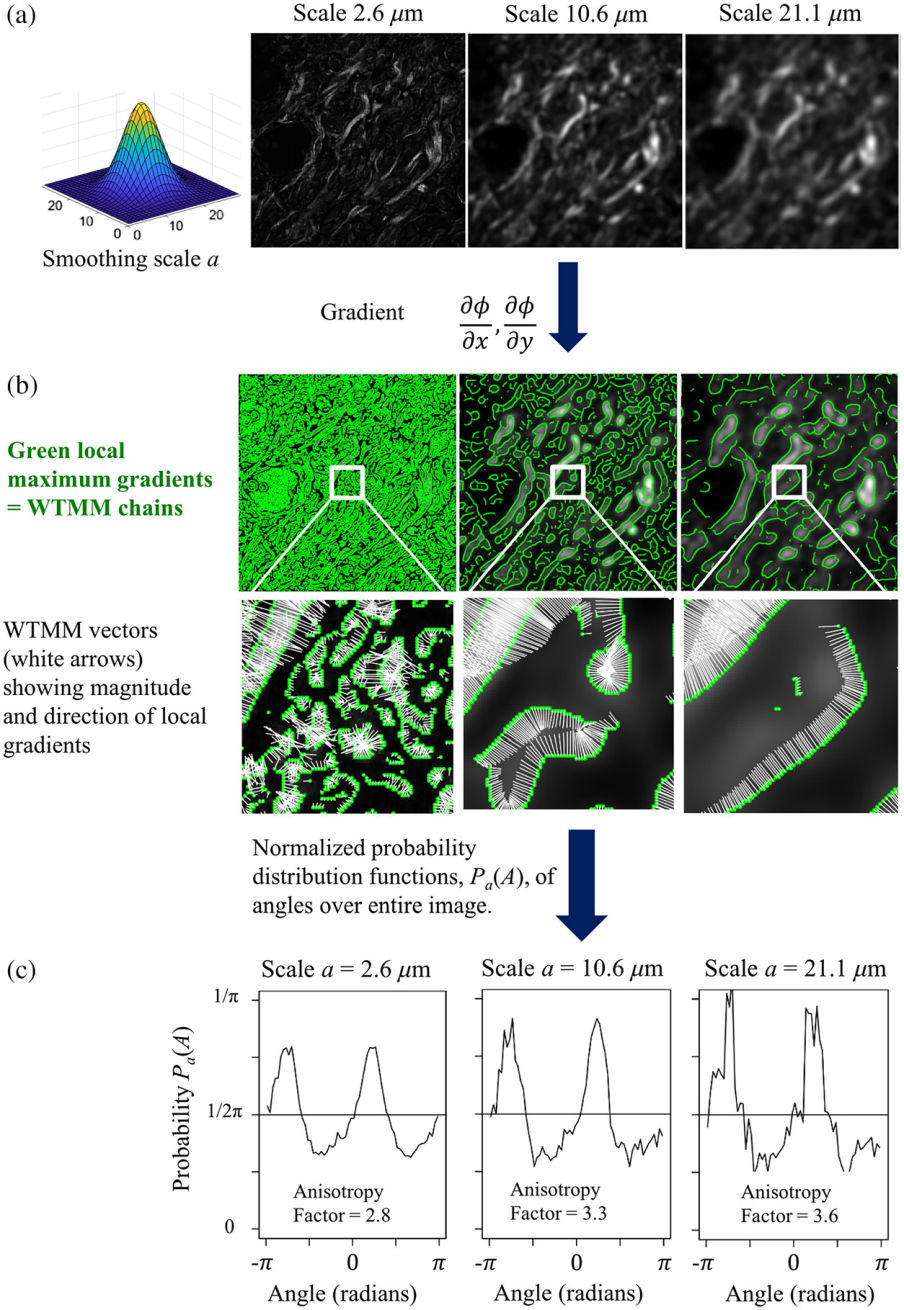
Flowchart of the 2D WTMM anisotropy method. (a) The original image is convolved with the wavelet functions at various size scales (three scales are shown here). (b) Positions in the image where the intensity gradient is locally maximum are given by the WTMM, which form edge detection contours. Each WTMM vector points in the direction of maximal gradient (highest modulus value). (c) The distribution of the angles of these vector directions is tabulated in pdfs, $P_{a}(A)$, from which the anisotropy factor, $F_{a}$ is calculated.

At a given scale a>0, the wavelet transform modulus maxima are the specific locations b within the image, where the modulus $M_{\psi }[f](b,a)$ is locally maximum in the direction of the argument $A_{\psi }[f](b,a)$. At each size scale a, these WTMM are automatically organized as “edge detection maxima chains.”^51^^–^^53^^,^^55^ Additional algorithmic details can be found in the Appendix of Ref. 65. The maxima chains for three different size scales are shown in green in Fig. 2.

An image having an anisotropic signature means that the intensity variation in the image will differ according to the direction considered. These images can be easily characterized from the directional information provided, at all size scales a, by the probability density functions (pdfs), $P_{a}(A)$, of the angles, A, associated to each WTMM vector (see Fig. 2). A flat pdf indicates unprivileged random directions of sharpest intensity variation (i.e., isotropy), while any departure from a flat distribution is interpreted as the signature of anisotropy. For the study of SHG imaging of collagen, a strong anisotropic signature is interpreted as a strongly structured collagen fiber lattice.^74^

### 2.6 Anisotropy Factor, $F_{a}$

To obtain quantitative information from the angle pdfs $P_{a}(A)$, they are compared with a theoretical flat distribution representing an ideal isotropic signature. The anisotropy factor, $F_{a}$, defined for each value of the scale parameter a, is given by the area between the curve corresponding to the observed pdfs and a flat normalized distribution (i.e., a constant value of $\frac{1}{2\pi }$):

$$F_{a}=\int_{-\pi }^{\pi }|P_{a}(A)-\frac{1}{2\pi }|dA.$$

Therefore, a theoretically isotropic surface will have a pdf of $P_{a}(A)=\frac{1}{2\pi }$ and therefore an anisotropy factor value of $F_{a}=0$, while any value greater than 0 quantifies a departure from isotropy. Thus, this formalism objectively provides a quantitative assessment of morphological structure. A step-by-step explanatory diagram is shown in Fig. 2.

### 2.7 Synthetic Images

**Fig. 3:**
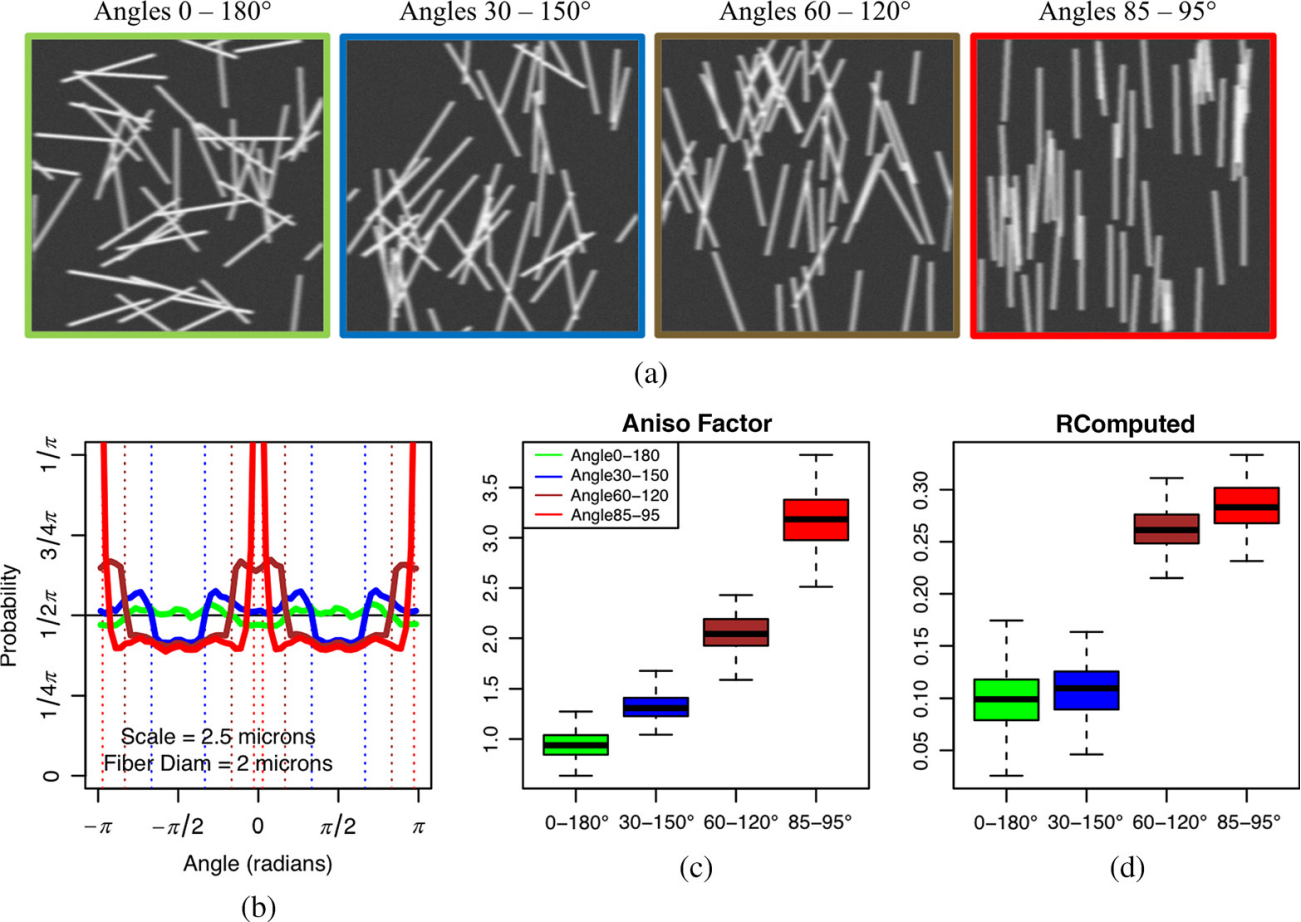
Calibration analysis on groups of 100 synthetic images, each containing 50 simulated fibers with 2  μm diameters. (a) Four sample synthetic images of fibers with alignment angles randomly taken from the ranges [0 deg, 180 deg] (green), [30 deg, 150 deg] (blue), [60 deg, 120 deg] (brown), and [85 deg, 95 deg] (red), respectively. (b) Average pdfs, $P_{a}(A)$, with a=2.5  μm. The dashed vertical lines mark the bounds of the ranges of angles theoretically expected (i.e., perpendicular to the fiber angles used in the simulations). (c) Corresponding boxplots representing the distribution of anisotropy factors, $F_{a}$, with a=2.5  μm, for each group of 100 simulations. (d) Comparative anisotropy analysis of the same datasets using the Fourier-based method yielding the $R_{\text{Computed}}$ factor.

Batches of synthetic images were created using a Matlab script previously described in Ref. 74. In each synthetic image, 50 simulated collagen fibers, represented as individual rectangles along the y-direction, were generated. In this work, the diameter of individual fibers was either 2, 4, 6, or 8  μm, which is representative of the range of collagen fiber diameters found in SHG imaging studies.^5^^,^^9^ The length of the individual collagen fibers was uniformly sampled from the range of 40 to 60  μm. Within a single image, the positions of the fibers were uniformly sampled across the entire simulated 512×512  pixel area modeling the SHG 180×180  μm FOV corresponding images. The angle of each fiber, θ, was uniformly sampled from a given range $\left[\theta_{\min},\theta_{\max}\right]$. Nineteen such ranges of angles were considered, from high alignment, $\left[\theta_{\min},\theta_{\max}]=[85\;\deg,95\;\deg\right]$ to no alignment, $\left[\theta_{\min},\theta_{\max}]=[0\;\deg,180\;\deg\right]$, in increments of 5 deg (i.e., [85 deg, 95 deg], [80 deg, 100 deg], [75 deg, 105 deg], …, [0 deg, 180 deg]). Noise was simulated using a combination of a 2D circular averaging filter with radius of 3 and motion filters in Matlab.^74^ Fibers were allowed to intersect in all cases. In total, 7600 images were simulated to calibrate the WTMM anisotropy method and to compare its performance with a Fourier-based angular amplitude approach: 100 images per angle range and for each diameter (100×19×4=7600). Sample synthetic images are shown in Fig. 3(a).

### 2.8 Noise Experiment

**Fig. 4:**
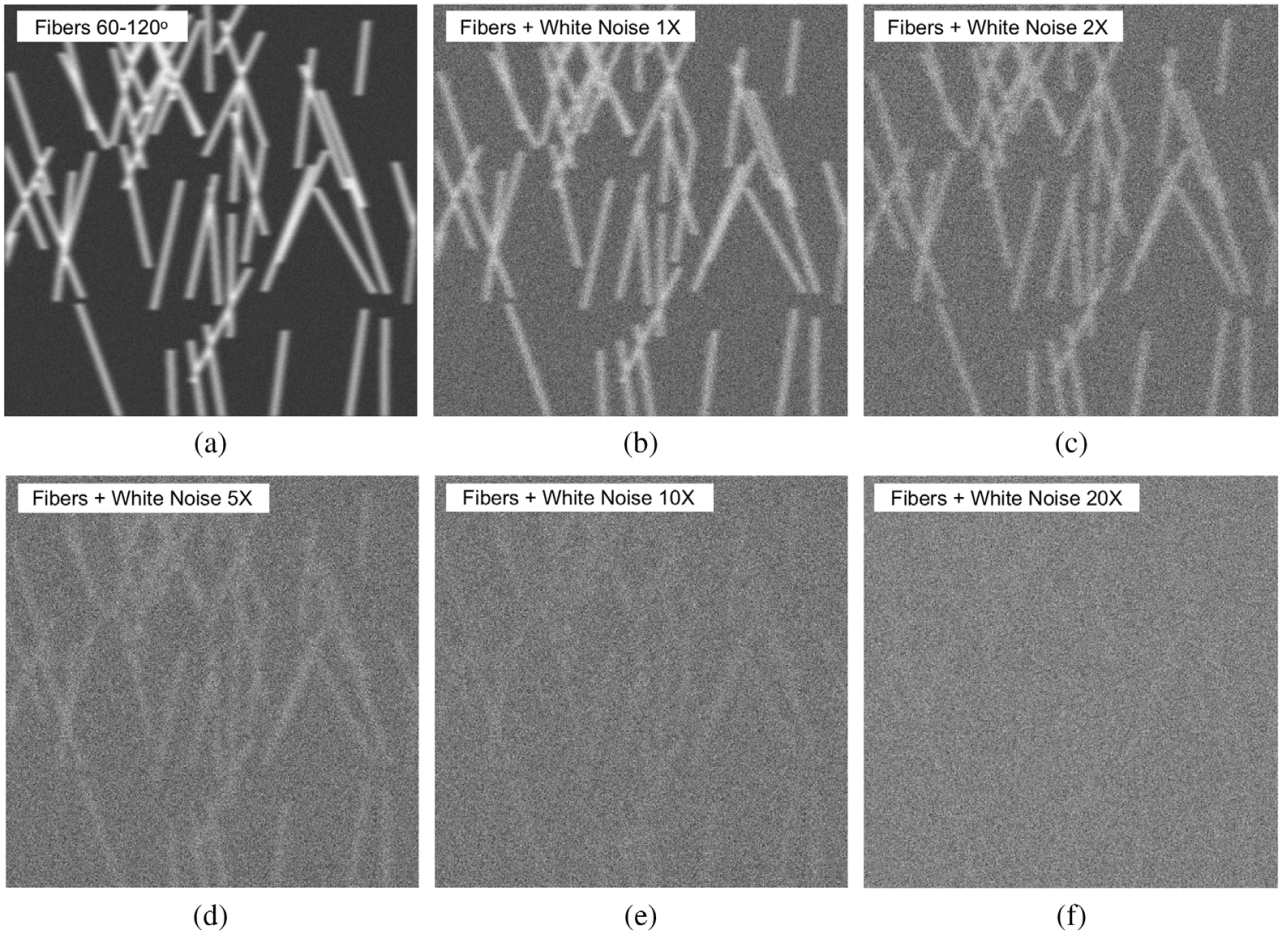
(a) Sample synthetic image of fibers with alignment angles randomly taken from the range [60 deg to 120 deg]. (b)–(f) White noise was added to the sample image in (a) at levels 1×, 2×, 5×, 10×, and 20×, respectively.

To test the robustness of the method and its capacity to properly evaluate the strength of the anisotropy in noisy conditions, increasing levels of white noise were added to a subset of synthetic images. In Fig. 4(a), a sample synthetic image as described in the previous subsection with fiber angles uniformly taken from the range $\left[\theta_{\min},\theta_{\max}]=[60\;\deg,120\;\deg\right]$ is shown. In Figs. 4(b)–4(f), increasing levels of white noise were added to the original image shown in Fig. 4(a) as follows: the mean pixel value from the original image in Fig. 4(a) was calculated. Then, a white noise image obtained through a white noise random simulator was generated with a mean pixel value that was a multiple of the original image. These multiples were 1, 2, 5, 10, and 20, respectively [Figs. 4(b)–4(f)]. For example, if the original image had a mean pixel value of 100, then the white noise image that was added to it in Fig. 4(f) had a mean pixel value of 100×20=2000. With high levels of white noise added to the original image, the fibers are buried under the very high pixel values from the noise that is added to it.

### 2.9 Fourier-Based Angular Amplitude Measurements: $R_{\text{Computed}}$

FT-based approaches have been used by several research groups to characterize morphological properties of collagen fibers.^45^^,^^74^ Recently, such a Fourier approach based on an angular amplitude measurement eliminated the need for fitting or thresholding, thus providing a fast and robust collagen fiber morphology assessment.^74^ The Fourier-based angular amplitude measurement is based on the characterization of fiber alignment in the Fourier domain. In short, a 2D image in the real domain can be Fourier transformed into the spatial domain, and by considering the amplitude of the 2D spatial frequency coordinates, the degree of collagen fiber alignment can be visualized. Images with high alignment will have nonuniform 2D spatial frequency amplitude patterns around the origin, whereas images with low alignment will have more uniform 2D spatial frequency amplitude patterns around the origin. To quantify the alignment in 2D Fourier space without any fitting or thresholding procedures, the content of the 2D spatial frequency amplitude is integrated at every angle from 0 deg to 180 deg. The sum of all vectors is then normalized by angular projection of all of the fibers within an image, which yields a value referred to as $R_{\text{Computed}}$, where 0 is random alignment and 1 is perfect fiber alignment. For more detail, see Ref. 74.

## 3 Results

### 3.1 Calibration Analysis

Groups of 100 synthetic images each containing 50 simulated fibers with diameters of 2, 4, 6, and 8  μm and with ranges of angles as described in Sec. 2 were generated. The 2D WTMM anisotropy analysis of four of these groups of images with fibers with 2  μm diameters and ranges of angles of [0 deg, 180 deg], [30 deg, 150 deg], [60 deg, 120 deg], and [85 deg, 95 deg] is presented in Fig. 3. Sample images for each angle range are shown in Fig. 3(a). Figure 3(b) shows the pdfs, $P_{a}(A)$ at an a=2.5  μm scale for each angle range (averaged over all 100 synthetic images for each group). Given that the WTMM vectors point toward the direction of largest gradient values (given by the modulus of the WT), their angles are perpendicular to the angle of the simulated fiber. Thus we observe very strong preferences for such perpendicular angles. For example, for the angle range [85 deg, 95 deg] (red), we expect the WTMM vectors to point preferentially toward the perpendicular, i.e., toward [−5 deg,5 deg] and [−175 deg,175 deg]. These expected perpendicular angle bounds are shown in dotted vertical lines in Fig. 3(b), which strongly coincide with the stepwise behavior of the distributions for each of the four angle ranges considered. For each group of 100 synthetic images, the distribution of anisotropy factors, $F_{a}$, is shown as box plots in Fig. 3(c), which shows a clear separation of groups from each of the four ranges of angles shown. As a comparison, the Fourier-based approach, which yields the $R_{\text{Computed}}$ [Fig. 3(d)], did not discriminate between the four groups of ranges of angles as well as the wavelet approach did.

### 3.2 Sensitivity Analysis

**Fig. 5:**
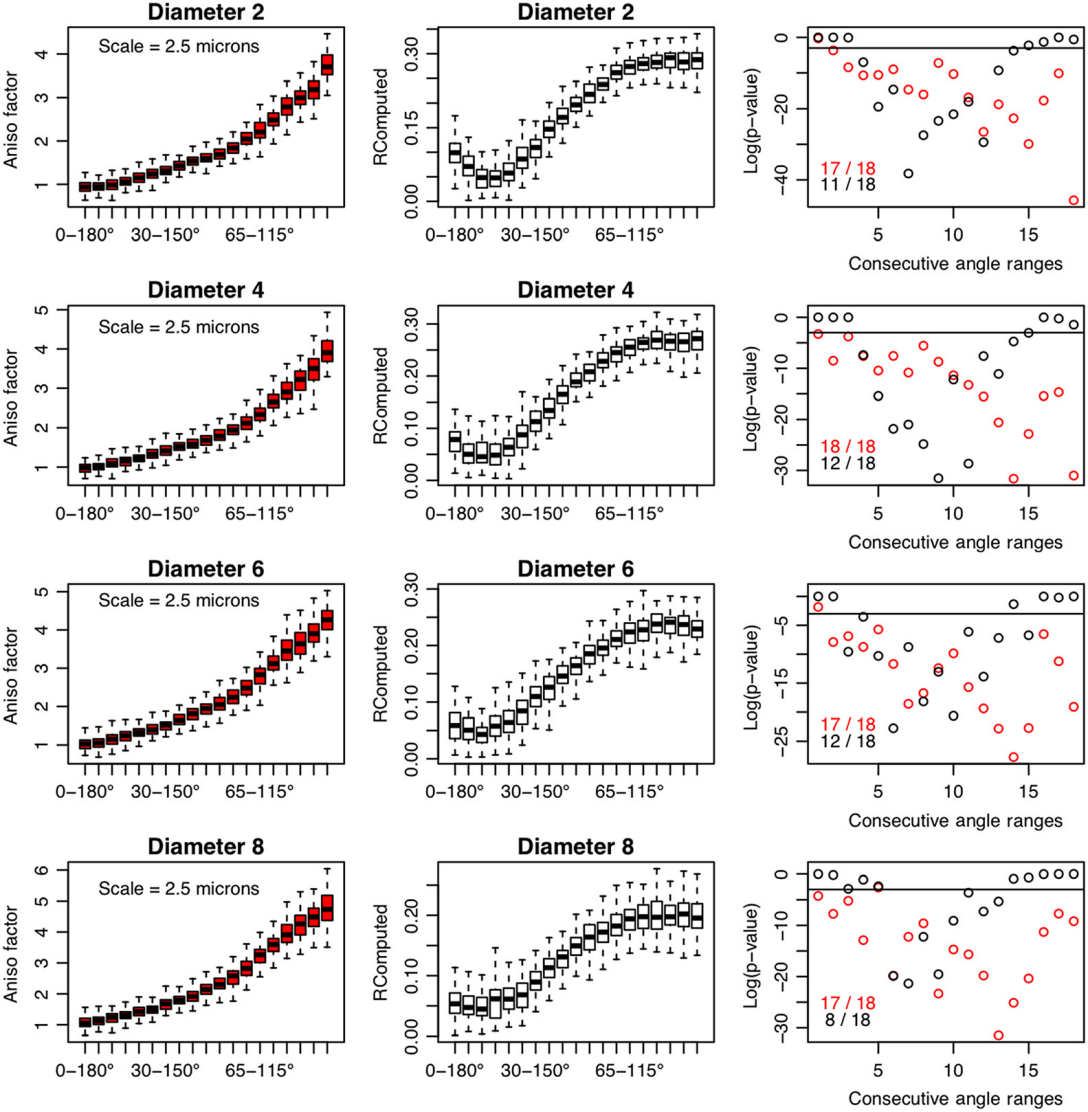
Deeper anisotropy calibration analysis on groups of 100 synthetic images each containing 50 simulated fibers. Compared with Fig. 3, where four ranges of fiber angles were considered, here 19 ranges were considered, from high alignment ([85 deg, 95 deg]) to no alignment ([0 deg, 180 deg]) in increments of 5 deg (i.e., [85 deg, 95 deg], [80 deg, 100 deg], [75 deg, 105 deg], …, [0 deg, 180 deg]). The boxplots representing the distribution of anisotropy factors, $F_{a}$, for each group of 100 simulations (left column) allow us to assess the sensitivity of the method to discriminating between consecutive ranges of angles. Similar results from the Fourier-based method are shown in the center column. In the right column, for each consecutive pair of angle ranges, each point plotted (red for the 2D WTMM anisotropy method, black for the Fourier-based method) corresponds to the p-value obtained from running a nonparametric Wilcoxon rank sum test. A p-value<0.05 indicates that the method was able to discriminate between groups of synthetic fiber images of consecutive angle ranges in a statistically significant manner. Also included in these plots are the total number (out of a possible 18) of consecutive angle ranges for which each method was able to successfully discriminate. The scale used here is 2.5  μm.

To further investigate the sensitivity of the 2D WTMM anisotropy method to discriminating between synthetic images of simulated fibers with different ranges of directional angles, 19 groups of different ranges of angles were analyzed, as described in Sec. 2. This deeper analysis, presented in Fig. 5 (for scale 2.5  μm), provides further demonstration of the robustness of the wavelet approach. The 2D WTMM anisotropy method is capable of discriminating between groups of synthetic images with consecutive ranges of angles (e.g., between images with fibers taken from the range [0 deg, 180 deg] versus images with fibers taken from [5 deg, 175 deg], etc.). At a 2.5  μm scale, the method was able to provide statistically significant discrimination 17 out of 18 times for simulated fibers with diameters of 2, 6, and 8  μm, and 18 out of 18 times for simulated fibers with diameter 4  μm diameters. In stark contrast, the Fourier-based method did not perform as well: 11/18, 12/18, 12/18, and 8/18 for diameters 2, 4, 6, and 8, respectively. To be fair, one should investigate whether the Fourier-based method might perform better or worse depending on the spatial frequency used. Nonetheless, to provide a more in-depth exploration of the sensitivity of the wavelet approach, a similar analysis as shown in Fig. 5 was done using 40 different size scales, from 2.5 to 39.4  μm (data not shown). Across these 40 size scales, the median number of consecutive angle ranges for which the 2D WTMM anisotropy method was able to successfully discriminate is: 17/18 for simulated fibers with diameters of 2, 6, and 8  μm, and 18/18 for fibers with 4  μm diameters. This provides further evidence that, even if a Fourier-based method was used at different spatial frequencies, it seems unlikely that it would perform as well as the wavelet approach.

### 3.3 Noise Experiment

**Fig. 6:**
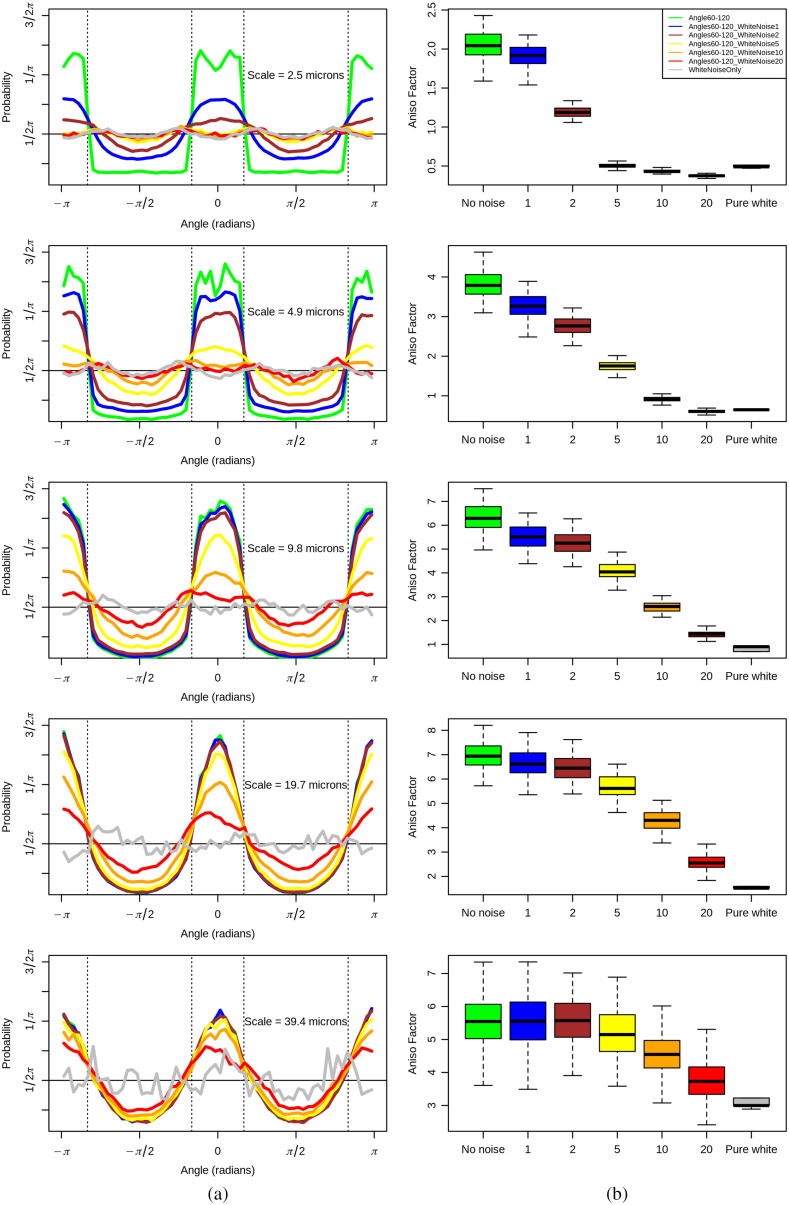
Application of the 2D WTMM anisotropy method on seven groups of 100 simulated images, at scales of a=2.5, 4.9, 9.8, 19.7, 39.4  μm. The seven groups of data correspond to the [60 deg, 120 deg] synthetic fiber simulations (no noise, shown in green), the five levels of white noise added to these synthetic images (shown in blue, brown, yellow, orange, and red; also see Fig. 4), and a white noise only group (shown in gray). (a) Average pdfs, $P_{a}(A)$. The dashed vertical lines mark the bounds of the range of angles theoretically expected (i.e., perpendicular to the fiber angles used in the simulation: [60 deg, 120 deg]). (b) Corresponding boxplots representing the distribution of anisotropy factors, $F_{a}$, for each group of 100 simulations and for each level of noise.

As shown in Fig. 6, as long as we consider a large enough scale (keeping in mind that pure white noise is a small-scale phenomenon), the analysis becomes blind to the noise and still adequately captures the anisotropic signature caused by the alignment of the fibers. This is still true even when white noise is added at 20× the amplitude of the original fiber image, albeit with a lower anisotropy factor than when there is less noise. Interestingly, when we calculate the anisotropy factor for pure white noise only (which is known to be isotropic, so its associated anisotropy factor is close to zero), it is very significantly different than the fibers with +20× amplitude white noise at large wavelet scales ($p\text{-value}<10^{-16}$ for scale 39.4  μm), even though visually it is hard to see the fibers under the 20× noise.

### 3.4 Anisotropy Analysis of Mouse Skin SHG Images

**Fig. 7:**
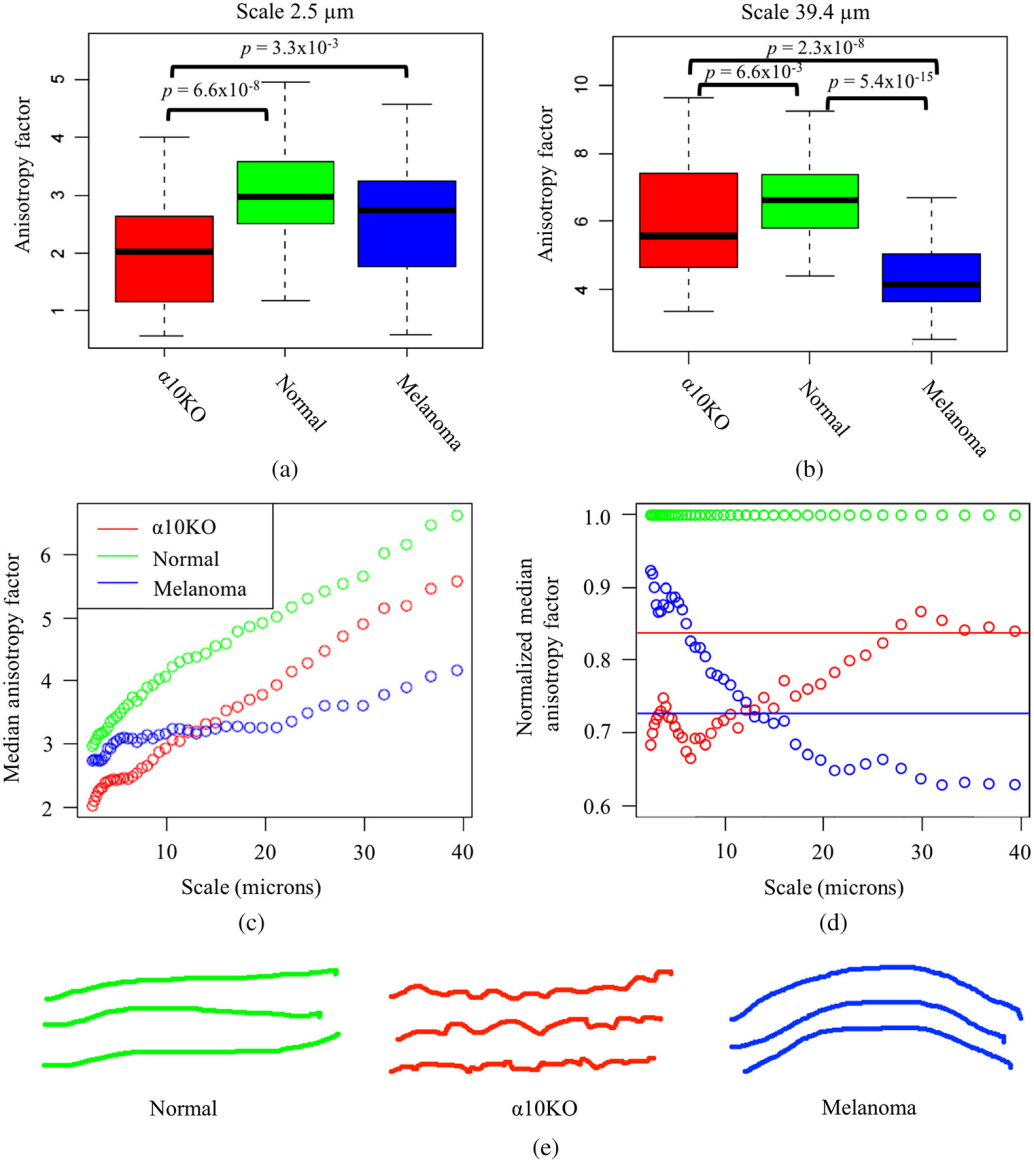
Multiscale anisotropy analysis on SHG images of mouse skin: α10KO (red), normal (green), and melanoma (blue). Box plots representing the distribution of anisotropy factors for the three groups of skin samples at (a) the smallest scale considered (2.5  μm) and b) the largest scale (39.4  μm). All of the intermediate scales in between are shown in (c). To observe similarities and differences between the α10KO and melanoma skin images versus the normal skin images, the median values in (c) were normalized by dividing by the normal skin median values (d). Also shown in (d) are the analogous normalized values for $R_{\text{Computed}}$, represented only as horizontal lines (red for α10KO and blue for melanoma) because only one spatial frequency was used with this method. Exaggerated cartoons depicting proposed collagen fiber structures are shown in (e).

Cohorts of images from the mouse skin SHG images listed in Table 1 were analyzed with the 2D WTMM anisotropy method. The box plots shown in Figs. 7(a) and 7(b) correspond to the anisotropy factors, $F_{a}$, for these three cohorts at scales of a=2.5 and a=39.4  μm, respectively, i.e., the smallest and largest scales considered. Drastically different anisotropy signatures are observed at these two extreme scales. Interestingly, there seems to be a somewhat monotone transition between these two extreme behaviors as a function of scale, as shown in Fig. 7(c), where all scales between a=2.5 and a=39.4  μm are shown. To provide a relative comparison of the melanoma stimulated and the integrin α10KO skin collagen fibers when they are each compared with normal skin, the data in Fig. 7(c) were normalized by dividing every value by the value obtained for normal skin, as shown in Fig. 7(d). Also shown in Fig. 7(d) are the analogous normalized values for $R_{\text{Computed}}$, represented only as horizontal lines (red for α10KO and blue for melanoma) because only one spatial frequency was used with this method.

#### 3.4.1 Melanoma stimulated versus normal skin

At an a=2.5  μm scale [Fig. 7(a)], the anisotropy factors for the melanoma stimulated skin SHG images are not statistically significantly different than the normal skin images. However, at an a=39.4  μm scale [Fig. 7(b)], the difference becomes extremely significant ($p∼10^{-15}$), showing that at large scales the alignment of the melanoma stimulated skin collagen fibers is more random (lower $F_{a}$) versus the more aligned collagen fibers (higher $F_{a}$) of the normal skin images.

#### 3.4.2 Normal skin versus integrin α10KO skin

At all scales considered, the normal skin collagen fibers are more aligned (higher $F_{a}$) than the integrin α10KO skin collagen fibers [Figs. 7(a)–7(c)—green versus red]. This difference is statistically significant for all scales considered. However, there is a trend [best seen in Fig. 7(d)] showing that the integrin α10KO skin collagen anisotropy factors, $F_{a}$, start to approach those of the normal skin at larger scales.

#### 3.4.3 Melanoma stimulated versus integrin α10KO skin

There is a reversal of behavior when comparing the melanoma stimulated versus integrin α10KO skin anisotropy factors, $F_{a}$, from small scales to large scales. At small scales, the melanoma-stimulated skin collagen fibers are more aligned than the integrin α10KO skin collagen fibers [Fig. 7(a)], but at large scales, it is the opposite [Fig. 7(b)]. For the scales in between, there is a transitory inverse behavior from small to large scales [Figs. 7(c) and 7(d)], with a crossover at an a∼15  μm scale.

Taken together, the comparisons in anisotropy at different size scales for the melanoma stimulated, integrin α10KO, and normal skin collagen SHG images are represented by a cartoon representation shown in Fig. 7(e). In this simplified hypothetical scenario, the normal collagen fibers (green) would be mostly straight and aligned, regardless of the scale considered. In contrast, the melanoma-stimulated skin collagen fibers (blue) would be relatively similar to the normal skin collagen fibers at small scales, but quite different at large scale. And conversely, the integrin α10KO collagen fibers would be relatively similar to the normal skin collagen fibers at large scale but quite different at small scale.

## 4 Discussion and Conclusions

In this paper, we present a powerful 2D multiscale wavelet-based image analysis tool that allows for robust and sensitive anisotropy measurements of formalin-fixed collagen fibers in mouse skin. The 2D WTMM anisotropy method, which was first introduced for the study of astrophysical maps^56^ and then to study muscle cell morphology in zebrafish^67^^–^^71^ and soft tissue in-growth into artificial bone implants,^57^ was adapted and presented here as a technique to study the alignment of collagen fibers from SHG images of skin samples. The use of formalin-fixed tissues introduces new crosslinking; however, based on prior imaging studies using SHG, there is minimal impact of formalin fixation on collagen fiber orientation.^75^ Furthermore, the 2D WTMM method is restricted to gradient changes within the image due to resolvable features (∼700  nm) which are much larger than the alterations introduced from the addition of formalin induced crosslinks (30 to 70 nm size scale).

### 4.1 Importance of Multiscale Analyses

Critically important observations can be missed when using a tool that only considers a single scale (or a single frequency, if using a Fourier-based method). Clearly, it would be impossible to observe the drastically different behaviors at different size scales that are presented in this study. For example, if a single-scale analysis had been performed on these data at an a∼15  μm scale [Figs. 7(c) and 7(d)], then it would have been impossible to discriminate between the melanoma-stimulated and integrin α10KO skin collagen fibers. This would have forced the analyst to report inconclusive results. But perhaps even more importantly, suppose two different analysts were studying these data at two different individual scales, say analyst 1 at one given scale a<10  μm and analyst 2 at a given scale a>20  μm. Then, the two analysts would have reached contradictory conclusions. Analyst 1 would conclude that melanoma-stimulated skin collagen fibers are statistically significantly more aligned (higher anisotropy factor $F_{a}$) than integrin α10KO skin collagen fibers and analyst 2 would report the exact opposite.

We hypothesize that the superiority of the 2D WTMM anisotropy method is due to the wavelet transform’s ability to analyze both space and scale (analogous to time and frequency for signal processing), compared with Fourier techniques being limited only to scale (frequencies). There are drawbacks that come with this additional analytical power associated with the WTMM method, namely algorithmic complexity and higher computation time, versus the simpler and faster FT.

### 4.2 Biological Interpretation

Given the sensitivity of the 2D WTMM method to detecting specific differences in collagen fiber orientation over multiple size scales, this important technique allows for a more precise analysis of subtle changes in collagen organization that may play functional roles in governing cellular behavior *in vivo*. To this end, previous evidence suggests that collagen fiber spacing, density, and orientation play important roles in regulating tissue morphology, cell shape, and integrin-mediated signaling that collectively helps control cell behavior.^10^^,^^26^^,^^76^^–^^79^ For example, changes in collagen architecture have been shown to modulate integrin binding and promote both normal and pathological processes such as wound healing, angiogenesis, and tumor growth and metastasis.^10^^,^^26^^,^^76^^–^^79^ However, while considerable insight is available as to how collagen binding integrins can control gene expression,^24^^,^^25^^,^^29^ much less is known regarding whether collagen-binding integrins play roles in regulating the orientation of collagen fibers. In this regard, while integrin α10 appears to play a role in regulating collagen network abundance in murine bone,^35^ our new studies now suggest that α10 integrin may also contribute to the control of collagen fiber orientation, as distinct differences were observed between collagen fiber orientation within normal skin and skin from mice in which integrin α10 was knocked out.

Integrins including α1β1, α2β1 α10β1, and α11β1 are known to serve as functional cell surface receptors for collagen.^24^ Integrin-mediated binding of collagen can result in activation of multiple downstream signaling events leading to activation of effector molecules that control cytoskeletal dynamics including small GTPases such as RhoA and Rac as well as the transcriptional coactivator “Yes Associated Protein” (YAP), which collectively regulate the ability of fibroblasts and melanoma cells to apply tensional forces to collagen, leading to contraction and reorganization of fiber orientation.^80^^–^^82^ In fact, studies have revealed a potential role for the collagen-binding integrin α11 in controlling collagen orientation and contraction, as collagen fibers associated with breast tumors growing in transgenic mice lacking integrin α11 exhibited a more curved and disordered fiber orientation as compared with a straighter and more aligned collagen orientation found in mice that express this integrin.^83^ While little evidence is currently available concerning the ability of integrin α10 to alter collagen fiber contraction, studies have indicated that integrin α10 can be expressed in fibroblasts along with alpha-smooth muscle cell actin (αSMA) which exhibit characteristics of a highly contractile phenotype.^30^ Interestingly, studies have also indicated that inhibiting α10 integrin-mediated melanoma cell binding to denatured collagen can reduce the levels of active YAP^31^ and that reduced levels of activated YAP in fibroblasts can alter their ability to contract collagen.^81^^,^^82^ Given these studies, it would be interesting to speculate that the changes in the orientation of the skin collagen fibers observed in mice expressing integrin α10 as compared with mice lacking α10 might be associated with the altered ability of fibroblast lacking α10 integrin to efficiently contract the skin collagen into a straighter and more aligned orientation. In addition, given that both melanoma cells and fibroblasts can express α10 integrin^30^^,^^31^ and that these different cell types may bind to collagen at different positions on the fibers, along with studies indicating that these different cell types exhibit distinct levels of collagen contraction,^84^ when taken together, these parameters may contribute in part to the differential orientations observed between collagen from normal skin and collagen from skin stimulated with melanoma over the size scales studied.

The exact mechanism by which integrin α10 regulates collagen fiber orientation is currently not known, and further studies will be needed to elucidate the mechanisms that contribute to this surprising observation. However, developing more precise methods and strategies to accurately identify, characterize, and quantify changes in collagen orientation over multiple size scales *in vivo* may not only provide a better understanding of how integrins contribute to changes in collagen structure but also how biophysical changes in collagen organization *in vivo* regulate cellular behavior. Taken together, our studies, in conjunction with existing insight into how collagen orientation contributes to physiological and pathological processes, may ultimately not only help identify new therapeutic strategies for the treatment of diseases controlled by ECM remodeling but also help optimize the use of engineered collagen containing tissue scaffolds for multiple applications.

## Code, Data, and Materials Availability

Code and image data used in this manuscript can be made available by reasonable request to the corresponding authors.
